# Magnetotelluric support for edge-driven convection and shear-driven upwelling in the Newer Volcanics Province

**DOI:** 10.1038/s41598-023-32403-z

**Published:** 2023-04-04

**Authors:** S. Jennings, G. Heinson, D. Hasterok, B. Kay

**Affiliations:** Mawson Centre for Geoscience, University of Adelaide, North Terrace, SA 5005 Australia

**Keywords:** Geophysics, Geodynamics, Volcanology

## Abstract

Intraplate volcanic provinces present significant natural hazards to many populated regions globally but their origins are poorly understood. Though hypotheses involving mantle plumes are predominant, the Newer Volcanics Province of southeast Australia—a relatively young (< 4.5 Ma), EW trending collection of over 400 volcanic centres—is increasingly attributed to some combination of edge-driven convection (EDC) and shear-driven upwelling (SDU). In this paper, we provide magnetotelluric (MT) data in support of these geodynamic processes. Three-dimensional inversion of 49 new broadband MT sites, in combination with 143 previously collected broadband, long-period, and geomagnetic depth soundings, reveals an elongate zone of moderately low resistivity (∼ 10–300 Ω m) spanning the Mt Gambier subprovince at a depth of between 20 and 40 km. The newly defined Gambier Conductor is contiguous to, and orientationally aligned with, significant step in the seismically-defined lithosphere-asthenosphere boundary (LAB) presented by earlier studies. Moderately low resistivity is interpreted as fluid-catalysed alteration of iron-bearing crust resulting from percolating magmatic volatiles. We argue that localised low resistivity (< 10 Ω m) at ~ 25 km depth in the mid-lower crust is associated with 1.2–3.6% partial melt. Supporting evidence indicates possible crustal thickening from 5.8 Ma at a rate comparable to estimates of SDU-induced surface eruptions and previous NVP production rate estimates.

## Introduction

In light of the recent eruption of Hunga Tonga–Hunga Ha’apai, which captured global attention in late 2021 as it impacted the more than 100,000 inhabitants of the South Pacific island nation of Tonga, understanding the threat posed by unanticipated explosive eruptions is of critical importance. Within Australia, the threat of eruptions is largely contained within the Newer Volcanics Province (NVP) of southeast Australia, a young (4.5 Ma to present) continental intraplate volcanic province^1,2^ that encompasses the populous centres of Geelong, Ballarat and western areas of Melbourne.

**Figure 1:**
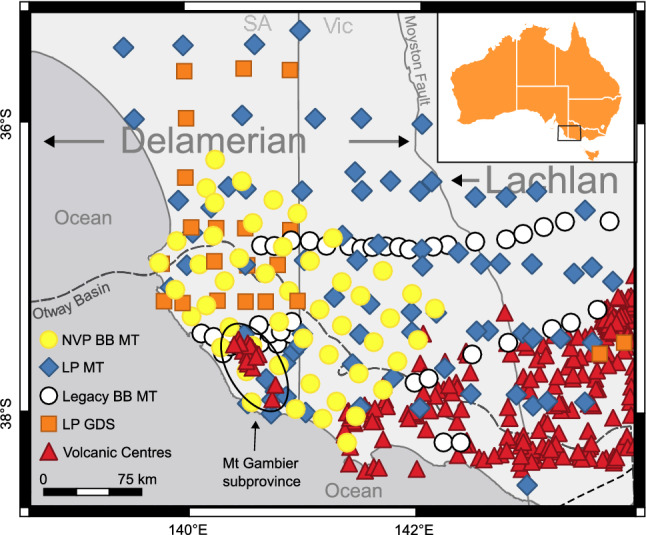
Tectonic setting of the study area displaying the major structural boundaries and outline of the Otway Basin. The east-dipping Moyston Fault represents a major crustal-scale boundary between the older Delamerian and younger Lachlan orogenic belts. The Otway Basin is a large, Late Jurassic rift basin that trends predominantly NW–SE and covers both onshore and offshore South Australia/Victoria as well as offshore Tasmania. Red triangles indicate volcanic centres of the Newer Volcanics Province^3^. The Mt Gambier subprovince (circled) can be further subdivided into the Mt Burr Group (NW) and the Mt Gambier/Mt Schank group (SE). All MT and GDS data shown were used in the 3D inversion. Yellow circles—broadband MT sites as part of this paper, white circles—previously collected broadband MT, blue diamonds—AusLAMP and other long-period MT, orange squares—geomagnetic depth soundings. Visualisation created using the free and open source QGIS v3.22.8 (https://qgis.org).

Over 400 volcanic centres across an area of 19,000 km^2^ have been identified^3,4^ that penetrate crust of both the Delamerian and Lachlan fold belts, as well as sediments of the Otway Basin (Fig. 1). With an estimated eruption frequency of 1:10,800 years^3^, the NVP is an active volcanic province with the most recent eruption occurring ∼ 5 kyr ago at Mt Schank within the Mt Gambier subprovince^5^. Geographically bound by faulting in the south^6,7^, the Central Highlands (CH) and Western Plains (WP) subprovinces make up the Victorian portion of the NVP and contain the vast majority of eruption centres. The younger Mt Gambier subprovince lies entirely within South Australia and is far smaller than its western neighbours in both area and number of eruption centres. While significant progress has been made with regards to identification and descriptive categorisation^3^, the deeper origins of the province remain widely discussed^8–15^.

The origins of intraplate volcanic provinces such as the NVP can be enigmatic and are frequently discussed in the literature. Mantle plumes originating from deep within Earth^16^ arguably remain the most popular explanation of intraplate volcanics but fail to explain those provinces with age distributions inconsistent with established plate motions and geochemistry that indicates an upper mantle source. In such cases, alternative explanations are favoured that are irrespective of age progression and directly invoke plate boundary processes such as subduction-related mantle return flow^17^, slab tear^18^, bursts in slab flux^15^ or removal/thinning of the lithosphere due to delamination^19^.

Many early hypotheses regarding origins of the NVP pursued the idea of one or more plumes beneath a northward migrating Australian plate^20–24^. However, established hotspot tracks that pass through the region^25^ are inconsistent with the E-W orientation of the NVP and ^40^Ar/^39^Ar age dating of NVP volcanics potentially indicates younging to the west, rather than south^13^. Additionally, observed seismic anomalies tend to terminate below ∼ 200 km, indicating an upper mantle source to be more likely than a plume^8^ which is in general agreement with petrogenetic modelling of basaltic volcanics within the province^26,27^.

Advances in regional seismic models reveal a complex sub-lithospheric topography in both the south and east of Australia^8,10,11,14^, leading to the relatively recent, but increasingly common (e.g.^8,9,13^), adoption of hypotheses involving a combination of EDC^28^ and SDU^29^. In the case of EDC, a thermal instability along a lithospheric step will produce a convection cell that may be further enhanced by long-wavelength thermal perturbations in the underlying mantle^28,30^. On the other hand, SDU promotes mantle upwelling through asthenospheric shear on viscosity heterogeneities^29^ or, more simply, the effect of lithospheric basal topography on relative asthenospheric flow due to a fast-moving Australian plate. Both the rate of upwelling and the location of the convection cell can be highly variable depending on the geometry of the step and the rate of plate motion^25^. Increasing the height of the step increases the rate of upwelling while a greater transition length—the horizontal distance between the shallowest and deepest extents—will push the cell further from the step. Increased plate motion (or relative asthenospheric flow) will enhance EDC and push the cell further from the step^25^; however, this is only the case if relative motion is perpendicular to the step and from thick to thin lithosphere^28^.

Alongside seismics, MT is one of few techniques capable of revealing lithospheric scale information regarding both the composition and structure of Earth. Previous regional MT studies include 2D inversion of 39 long-period MT stations which image a low-resistivity crustal feature (10–30 Ω m) beneath the CH and WP subprovinces attributed to ∼ 1.5–4% partial melt^31^. More recently, 3D inversion of compiled long-period MT and geomagnetic depth sounding (GDS) reveal the extent of the same conductor, but now attributed to deeply buried, carbon-rich sediments^32^. While Heinson et al.^32^ focus on a correlation with the overlying Victorian gold fields, they also reveal an independent, crustally-bound conductor (< 300 Ω m) beneath the Mt Gambier subprovince to the west. In this study, we increase resolution across this western crustally-bound conductor by supplementing 143 pre-existing broadband, long-period and GDS sites with a further 49 broadband MT sites collected at 25 km site spacing in a grid of approximately 200 by 125 km.

## Discussion

**Figure 2:**
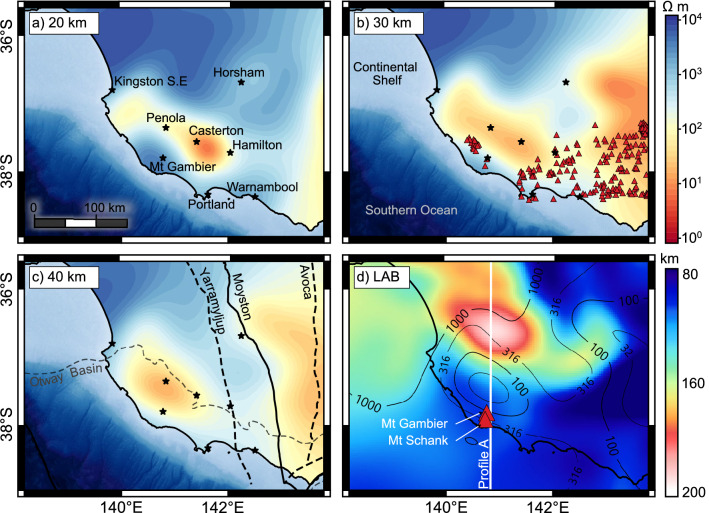
(**a**) resistivity at 20 km depth with overlying population centres. (**b**) resistivity at 30 km depth with volcanic centres of the Newer Volcanics Province^3^ (red triangles). (**c**) resistivity at 40 km depth with major crustal features and outline of the Otway basin. (**d**) depth in km to the seismically defined lithosphere-asthenosphere boundary (LAB)^14^ overlain with resistivity contours at 40 km depth. Profile A illustrates the cross-section from Fig. 3 and is aligned N-S approximately parallel to local plate motion (~ 356–003°N, relative to a fixed hotspot reference frame^33–35^). Visualisation created using the free and open source QGIS v3.22.8 (https://qgis.org).

Figure 2 illustrates resistivity depth slices at (a) 20 km, (b) 30 km and (c) 40 km, as well as (d) resistivity contours at 40 km depth superimposed on a background indicating depth to the seismically-defined LAB. Additional resistivity slices and further details regarding specific inversion parameters used during the modelling are provided in the Supplementary Material. Approximately 100 km north of the Mt Gambier subprovince, an imposing lithospheric keel extends to a depth of ∼ 190 km and grades rapidly to the south until it reaches a depth of ∼ 100 km beneath the volcanics.

At 40 km depth (Fig. 2c,d), a resistivity low of 16 Ω m exists approximately 90 km directly south of the lithospheric step. The resistivity structure at this depth closely mirrors the geometry of the northward step and is located downstream of relative asthenospheric flow—local plate motion is estimated between 356 and 003°N at ~ 8 cm/year relative to a fixed hotspot reference frame^33–35^. At depths greater than ~ 50 km, the conductor dissipates and eventually gives way to a general background resistivity. We note that below local Moho depths of approximately 32–40 km^36,37^, the MT method is unlikely to resolve small-scale conductive features related to vertical transport of magma.

**Figure 3:**
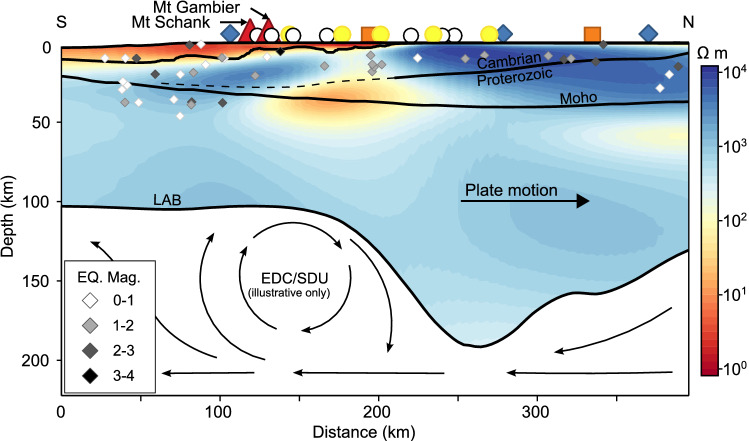
Profile A: N-S Resistivity cross-section passing close by Mt Gambier, Mt Schank, a resistivity low of ∼ 16 Ω m and a significant step in the seismically-defined lithosphere-asthenosphere boundary (LAB). Mantle flow vectors are not based on real data or modelling but are illustrative only. They serve to highlight relative mantle flow due to a northward moving Australian plate and the roughly anticipated position of a convection cell given the regional, seismically-defined LAB. The LAB^14^ and Moho^36,37^ surfaces are based on seismic modelling while the inferred Cambrian/Proterozoic interface^39,40^ is determined through gravity modelling. Yellow circles—broadband MT sites as part of this paper, white circles—previously collected broadband MT, blue diamonds—AusLAMP and other long-period MT, orange squares—geomagnetic depth soundings. Our model is masked below the LAB due to a focus on crustal structures and low resolution at sub-crustal depths. See the Supplementary Materials for a discussion on model resolution.

Moving up through the crust, the conductor maintains its orientation but becomes more extensive and increasingly elongate by 30 km depth (Fig. 2b). Between 20 and 40 km depth, the NW–SE orientational axis of the conductor shifts ∼ 17 km to the SW, consistent with potential fluid migration along an inferred^38^ NW–SE trending northern master fault of the Otway Basin. At 20 km (Fig. 2a), a resistivity low of < 10 Ω m is concentrated on the Victorian side of the border and attributed here to partial melt. Additional resistivity slices and cross sections in the Supplementary Material indicate that minimum resistivity occurs at approximately 25 km depth, which is shallower than magma reservoirs predicted below 32 km from spatial analysis of eruption point density^12^. Rising melt may stall in the mid to lower crust thanks to an inferred compositional boundary at the sub-horizontal interface between Proterozoic and Cambrian basement where a layer of oceanic crust/mantle material is interpreted to be intercalated with the tops of Proterozoic crustal blocks^39,40^ (Fig. 3). Such a layer would require more energy than the surrounding crust in order to induce partial melting and would therefore inhibit magma ascent.

Various two-phase mixing relationships were tested using the SIGMELTS 1.0 web-portal^41^ in order to obtain an estimate of potential partial melt fractions in the mid-lower crust at the depth associated with the point of minimum resistivity (~ 25 km). Conductivity of the melt phase was determined to be ~ 0.12 Ω m using an average of previously calculated parent melt compositions^27^ at a temperature of 1473 K. This temperature is high for typical mid to lower crust but significantly lower than estimated melt formation temperatures of 1706–1886 K^27^. Modelling the melt phase within a relatively resistive crust of 1000 Ω m, an estimated melt fraction of approximately 1.2–3.6% is determined in the mid-lower crust.

While resistivities of < 10 Ω m are attributed to partial melt, we suggest the greater Gambier Conductor (100–300 Ω m) is linked to percolating magmatic volatiles that are more easily transported and may therefore affect a wider region. Volatiles such as CO_2_ and S are not intrinsically conductive; however, they may alter the crust in such a way that resistivity may be observably reduced. For example, the presence of large quantities of CO_2_ can reduce resistivity through precipitation of interconnected grain-boundary graphite films, though such a scenario is unlikely due to the instability of graphite at high temperatures and would produce significantly lower resistivity than observed here^42^. Alternatively, fluid-catalysed oxidation of iron is expected to moderately enhance conductivity of iron-bearing rocks by increasing the ratio of Fe^3+^ to Fe^2+^ and thereby increasing the number of small polarons available for diffusion^43,44^. We therefore propose that the regionally extensive moderate resistivity found in the mid to lower crust beneath the Mt Gambier subprovince is the result of wide-spread volcanically induced metasomatic alteration of iron-bearing crust. Mid–upper crustal conductors recently imaged in the continental intraplate Tariat–Chuluut volcanic zone of central Mongolia were attributed to similar processes^45^.

Figure 3 presents a lithospheric scale cross-section through modelled resistivity along Profile A in Fig. 2, bisecting both Mt Schank and Mt Gambier, and aligned with the local trajectory of the Australian plate^33–35^. The lithospheric keel from Fig. 2 is observed here as a significant step immediately north of the Gambier Conductor and is expected to induce EDC/SDU processes immediately south, though the rate and location of upwelling will inevitably depend on the specific geometry and rate of plate motion^25,28–30^. For a hypothetical square step, SDU alone can account for a vertical upwelling rate of ∼ 2 cm per year^29^, though sloping basal topography will reduce the overall effect. The effect of plate motion on EDC depends on the velocity of relative asthenospheric flow. If relative flow is perpendicular to the step and from thick to thin lithosphere, as is expected in the presented scenario, greater velocity will enhance EDC and push the cell further from the step^25,28^.

**Figure 4:**
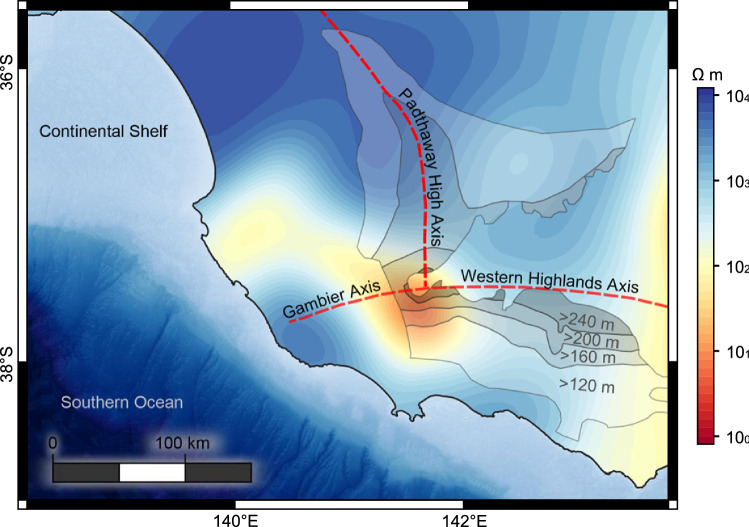
Strong spatial correlation between Cenozoic tri-axial uplift and the Gambier Conductor (20 km depth slice) alludes to localised crustal thickening related to magmatic processes of the Newer Volcanics Province. The resistivity slice has been clipped at the coastline as the model is unconstrained by data over the ocean. Dashed red lines represent three axes of uplift while semi-transparent contours are areas of known uplift occurring between 5.8 Ma and 3.0–2.5 Ma as identified by Wallace et al.^46^. From light to dark, these contours represent regions of 120–160 m, 160–200 m, 200–240 m, and + 240 m. Visualisation created using the free and open source QGIS v3.22.8 (https://qgis.org).

Figure 4 presents resistivity at 20 km depth overlain by three uplift axes^46^ interpreted to intersect ∼ 10 km north of the point of lowest resistivity. This area incorporates the Dundas Tableland and has been described previously as a broad domal structure of elevated topography surrounded by well-established radial drainage patterns^47,48^. Similar domal structures have been identified throughout the Western Highlands that are co-located with NVP volcanics^48^, though the closest volcanic centres to the axial intersection exist approximately 35 km to the south and east. The region features several faults that have reactivated throughout the Cenozoic^47^, during which time there is evidence for up to 240 m of uplift based on age dating and tracing of Pliocene strandlines^46^.

Uplift in southeast Australia is generally attributed to a NW–SE compressional regime beginning at ∼12 Ma^49^; however, given the spatial relationship between conductor and axial uplift, we introduce the idea that some portion of localised uplift may be the result of an emplaced layer of NVP basaltic material. In this case, localised uplift due to magma emplacement is interpreted as equivalent to the regional uplift (> 120 m) subtracted from a maximum uplift of ~ 240 m in the immediate area. The thickness of an emplaced layer of basaltic composition, ${h}_{l}$, that would yield uplift of 120 m can be estimated using the isostatic relationship,

$${h}_{l}=\Delta \varepsilon \left(\frac{{\rho }_{m}}{{\rho }_{m}-{\rho }_{l}}\right),$$

where $\Delta \varepsilon$ is the elevation change, ${\rho }_{m}$, the density of mantle and ${\rho }_{l},$ the density of the emplaced layer. Assuming a mantle density of 3330 kg m^−3^, 120 m of uplift can be explained by either a 1.72 km thick layer of crystallised gabbro (3100 kg m^−3^^50^) or 1.49 km using a density of 3065 kg m^−3^ for a layer consisting of 10% partial melt. In both cases it is noted that the effects of thermal buoyancy and/or mantle upwelling^51^ are neglected and therefore these numbers serve as an upper estimate. For an uplift period of approximately 3 million years^46^, these thickness estimates indicate an emplacement rate of 0.45–0.61 km Ma^−1^. Assuming emplaced magma would otherwise reach the surface, these numbers are consistent with proposed estimates of 0.16–1.3 km Ma^−1^ for surface eruptions attributable to SDU^29^. These emplacement rate estimates are also consistent with production rate estimates of 0.36 ± 0.22 km Ma^−1^ and ~ 0.39 km Ma^−1^ for Newer Plains basalts prior to 4 Ma^13^.

## Conclusions

We argue that the Gambier Conductor, typified by resistivity on the order of 100–300 Ω m, is the result of percolating magmatic volatiles that reduce resistivity via in-situ alteration of an iron-bearing mid to lower crust beneath the Mt Gambier subprovince of the NVP. The conductor is located contiguous to, and ‘downstream’ of, a significant lithospheric step, lending credence to hypotheses involving EDC and/or SDU processes in the upper mantle that give rise to basaltic magmas of the NVP. Lowest resistivity (< 10 Ω m) occurs at approximately 25 km depth and 65 km ENE of the Mt Gambier volcanics and is interpreted as 1.2–3.6% partial melt. This localised feature appears coupled with an inferred interface between Proterozoic and Cambrian basement that may impede surface eruptions and promote emplacement of magma at depth. Spatial correlation between the conductor and observed tri-axial uplift may indicate localised crustal thickening directly related to the NVP and at a rate comparable with SDU-based eruption estimates. The unanticipated location of partial melt outside the confines of the presently defined NVP is an important finding regarding understanding and mitigation of the risks involved with an active volcanic province for an expanding population in southeast Australia.

## Supplementary Information

Supplementary Information.

## Data Availability

The datasets generated and/or analysed during the current study are available via the South Australian Resources Information Gateway (SARIG). https://map.sarig.sa.gov.au.
