# Supplementary material for: Magnetotelluric support for edge-driven convection and shear-driven upwelling in the Newer Volcanics Province
Source: Sci Rep. 2023 Apr 4;13:5543. doi: 10.1038/s41598-023-32403-z (PMC10073071; doi:10.1038/s41598-023-32403-z)
Supplement: Supplementary file 1 — Supplementary Information. [file 41598_2023_32403_MOESM1_ESM.docx]

# Magnetotelluric support for edge-driven convection and shear-driven upwelling in the Newer Volcanics Province

**S. Jennings^a, ∗^, G. Heinson^a^, D. Hasterok^a^, B. Kay^a^**

*^a^Mawson Centre for Geoscience, University of Adelaide, North Terrace, SA, 5005, Australia*

Supplementary Information

# Geological Background

The broader tectonic environment can be traced back to the mid-Cambrian where stress transfer to the trailing edge of the Gondwana supercontinent from ~514 to 490 Ma formed the Delamerian Orogeny [1]. The roughly 24 Myr compressional event was characterised by Andean-style subduction of a westward dipping slab and was eventually terminated by rapid uplift, cooling and extension alongside late-stage granitic magmatism [1, 2]. The Delamerian Orogeny is the oldest part of the Tasman Fold Belt which forms the continental crust of eastern Australia [3, 4, 5] and exposure of basement rocks is largely confined to the west (main text, Figure 1). While much of the central region is covered by younger sedimentary sequences including the Otway Basin, magnetic marker beds related to the basaltic Truro volcanics reveal the eastward extent of the wider underlying province [6]. The major lithospheric structure of the Moyston Fault is interpreted as the boundary between the Delamerian and the younger Lachlan Fold Belts in the east [7, 8]. While the Moyston Fault has been shown to extend to the base of the crust [9], it is an eastward dipping structure and therefore should not bear significance with regards to our survey.

340 million years post cessation of the Delamerian Orogeny, the breakup of eastern Gondwana led to the separation of Australia and Antarctica [10] and development of the Australian Southern Rift System. This resulted in the formation of a number of Late Jurassic rift-basins along the south coast of Australia including the Otway Basin [11, 12, 13]. The Otway Basin is a large (~151 000 km^2^), predominantly northwest trending basin that covers both onshore and offshore South Australia and Victoria as well as offshore Tasmania.

The framework and history of the Otway Basin is described in great detail by numerous sources and encompasses multiple periods of rifting followed by thermal subsidence, accompanying basin sag and further sedimentation [14, 12, 15]. Deposition of fluvio-lacustrine sediments during the initial rift-subsidence phase led to formation of the Crayfish sub-group and the Eumeralla Formation [15]. The Crayfish sub-group consists mainly of interbedded sandstone and siltstone with less common shale, mudstone and claystone that can feature both chlorite and kaolinite [16]. The more widespread Eumeralla Formation overlies the Crayfish sub-group and is characterised by interlayered chloritic, micaceous and carbonaceous claystone and volcaniclastic and feldspathic sandstone [15]. Sediments generally thicken oceanward (SW) over the onshore portions of the basin, though numerous troughs and ridges defined by often crustal-scale, NW-SE trending faults characterise the basement topography.

# Structural control of the Gambier Conductor

Basement structure mostly in western Victoria has been modelled using a combination of gravity, magnetics and seismic data [17, 18, 19]. A deep seismic survey was first to image the Apsley Fault, an east-dipping, crustal-scale structure that extends to the Moho and is thought to be related to the Neoproterozoic breakup of Rodinia [9]. A number of regionally significant tectonic events that may have further altered basement structure since the Neoproterozoic. For example, rifting related to the breakup of Gondwana (and formation of the Otway Basin) is likely to have caused large scale basement faults that have been suggested as ideal pathways for NVP volcanics [20].

Recently, the Geological Survey of Victoria provided a model of the Northern Otway master fault [18] which aligns remarkably well with the structure of the Gambier Conductor (Supplementary Figure 1). The aforementioned Apsley Fault appears to constrain the northern limits of the conductor while the Hummocks Fault cuts through the least resistive regions. The lengthwise orientation of the Gambier Conductor is approximately 120 degrees east of geographic North, which is in good agreement with the roughly NW-SE orientation of initial depocenters of the Otway Basin and suggests alignment of the conductor is controlled by Otway structures. While the orientation of the conductor remains constant, the axis shifts NE by ~17 km between a depth of 40--20 km, indicating a dip of ~50° which is well within reason for rift-related normal faulting.

A sub-horizontal feature between the Proterozoic lower and Cambrian upper crust is modelled from a long-wavelength gravity high and inferred to be a high density layered unit [17]. The exact nature of the layer is uncertain but it is inferred to be oceanic crust/mantle material intercalated with the uppermost portion of the deeper Proterozoic blocks. Supplementary Figure 1 indicates that the Gambier Conductor is strongly coupled with this high-density layer and that lowest resistivity appears in close proximity to the thickest section. A dense mafic-ultramafic layer in the mid-crust provides a significant barrier to ascending magma of basaltic composition as such a layer will have a higher solidus temperature and require more energy to induce partial melting than surrounding country rock. This effective barrier means that rising magma may preferentially be emplaced in the crust rather than ascending to the surface.

# Penetration through the upper crust

The mid-crustal barrier described above may impede melt from penetrating into the upper-crust in large quantities; however, volatiles are more mobile and therefore more likely to pass through. Supplementary Figure 2 presents a structural cross section through the Penola Trough; a region of high potential for oil and gas exploration with several major discoveries including a commercial CO_2_ deposit. Isotopic analyses of noble gases from several wells in the region confirm a magmatic origin for CO_2_ and there are reports of recharging in some deposits [21, 22, 23, 24]. Percolating magmatic volatiles do not carry a net charge and will therefore be undetectable to MT soundings; however, gases may alter the country rock in such a way as to observably reduce resistivity.

Large quantities of CO_2_ can markedly reduce resistivity through precipitation of interconnected grain-boundary graphite films [25]; however, it does not appear likely in the present scenario given only moderate reductions in resistivity. H_2_O can moderately reduce resistivity of Fe-bearing rocks by increasing the ratio of Fe^3+^ to Fe^2+^, which increases the number of small polarons available for diffusion [26, 27]. The average H_2_O content of calculated parent magma compositions for Quartenary basalts within the Mt Gambier subprovince is reported as 5.18 wt% [20] so degassed water has theoretically passed through the crust in large quantities and at high temperature. We suspect that fluid-based alteration of Fe-bearing country rock resulting from percolating magmatic volatiles produces the conductive pathway through the upper crust seen in Supplementary Figure 1 and 2. The existence of the pathway itself is tested via additional 2D modelling in a later section.

Residence time for magma in the upper crust (based on modelling of erupted samples) is expected to be as little as one to ten days [20] which indicates a more direct pathway is required for melt to reach the surface. This suggests that significant tectonic movement is required along crustal scale structures in order to promote more rapid ascent. Structural evidence of inversion tectonics in the Otway Basin combined with analysis of earthquake focal mechanisms and in situ stress (borehole breakouts) indicates a NW-SE compressional regime with maximum horizontal stress at approximately N150° [28, 29, 30]. Such a stress regime would be favourable for reopening of NW-SE aligned structures in the region which could provide the pathway for melt to reach the surface.

# Data acquisition, processing and quality control

Over the course of two weeks in August 2019, 49 broadband MT sites were collected across the Mt Gambier region of the NVP using AuScope instruments (main text, Figure 1). The sites were set out in a rectangular grid trending NW-SE in parallel with the local coastline, approximately six rows deep and nine columns wide at ∼25km site spacing. The total area covered by the newly collected sites amounts to ∼25 000 km^2^. Sites were generally left to record data at 1000 Hz for a minimum of 40 hours (two nights) where possible; however, logistical constraints saw some sites fail to achieve this benchmark while others far exceeded it.

The survey region is within a moderately populated area with, in some places, significant cultural noise related to powerlines, main roads, farming and livestock. In almost all cases, 50 Hz powerline noise was evident to some degree but was removed along with accompanying harmonics using a -3 dB infinite-length impulse response (IIR) notch filter [31]. We greatly improve signal-to-noise ratio by using a robust processing algorithm referenced to a dedicated remote (site NVP-C5) specifically chosen for its low noise environment. Except for a ten-minute battery change after seven days, the remote site recorded data continuously throughout the duration of the survey. The resulting broadband MT dataset mostly encompass a period range between 0.01 and 1000 s where shorter periods relate to shallower depths.

Analysis of the phase tensor ellipses (Supplementary Figure 3) can provide useful information on dimensionality, strike and broader resistivity trends [32]. Sites to the south, confined by the bounds of the Otway Basin, display increasing conductivity at shorter periods (0.01 - 10 s) and indicate a shallow conductor in this region (Supplementary Figure 3b). The low skew angle and circular nature of ellipses within the basin at shorter periods (Supplementary Figure 3a, b) indicate the data is 1-D and consistent with previous resistivity studies in the region [33].

Outside the basin, ellipses are typically multi-dimensional, i.e., asymmetric, varying with depth and moderate skew. At 10 s and 300 s (Supplementary Figure 3c, e), skew angle increases for most sites indicating higher degrees of dimensionality at depths. At 100 s (Supplementary Figure 3d), sites within the bounds of the Otway Basin are becoming more resistive while outside the basin they become increasingly conductive. Just over half the sites reach a period of 1000 s (Supplementary Figure 3f), by which time the phase tensor ellipses are beginning to align in a NE-SW orientation. This broadscale geoelectric strike is most likely a coastal effect [34].

# Modelling

The starting model for our inversions included both an inner mesh, defined by the newly collected sites, and an outer mesh designed to encompass numerous sites from previous studies. The boundary of each mesh is visible in Supplementary Figure 3. The reasoning behind an outer mesh is to better define the boundary conditions of our inversion results by incorporating real data outside our region of interest. The inner mesh has cell spacing of 3000 m and extends 10 km beyond the edge of the sites collected in this study. The outer mesh has cell spacing of 5000 m and extends 30 km beyond the coastline to the southwest and 20 km beyond the sites collected in this study in all other directions. Beyond the outer mesh, 800 km of padding cells were added at a growth factor of 1.4. In the vertical direction, cell sizes start at 25 m and increase by a growth factor of 1.1 for the first 1000 m, followed by 1.06 for the next 100 km and finally by 1.2 until a maximum depth of 800 km is reached. In total, the starting mesh consists of just under 2.2 million cells.

With an increasing number of regional MT models being released, thanks largely to government funded initiatives such as AusLAMP, and a future emphasis targeted towards scale-reduction, it makes sense to incorporate information from regional modelling into our starting model. We therefore interpolate values from the regional model of Heinson et al. [35] onto our newly defined and finer spaced starting mesh. After doing so, we reassign a value of 0.3 Ω *m* to all ocean water cells to account for the change in cell size along the Australian coastline.

Our processed data were interpolated to a period range spanning 0.006 to 10,000 s, accounting for 32 periods over 6 decades that encapsulates both the broadband and long-period data used in the final model. It is noted that not all sites cover this full period range. For broadband sites, the period range typically extends from 0.01 s to a maximum of 1000 s and approximately half achieve less than 300 s. Long period sites generally range from ~10 to 10,000 s depending on quality.

A number of inversions were performed over the course of modelling using both the freely available ModEM inversion algorithm [36] and the proprietary 3-D inversion code provided by CGG Geotools™. Various input parameters including smoothing, damping and the inclusion of a priori information, all of which have been demonstrated to have variable effect on model results [37], were tested throughout the process. Our preferred model converged at an RMS of 1.45 using the CGG inversion algorithm with a horizontal smoothing factor of 1, a vertical smoothing of 0.1 and error floors of 5% for the impedance tensor. Data fits for select sites across the survey area can be examined in Supplementary Figure 4 and depth slices are presented Supplementary Figure 5.

## Model robustness

To assess the robustness of the 3D model, multiple inversions have been run to test the differences in horizontal and vertical smoothing weights, and near-surface smoothing. Supplementary Figure 6 shows four models for a depth slice of 20 km, and Supplementary Figure 7 for 30 km depth, with models (a) to (d) reducing horizontal and vertical smoothing weights allowing the final models to be rougher in both horizontal and vertical dimensions. RMS misfits decreases from (a) 2.14, (b) 2.03, (c) 1.83 and (d) 1.45. Model (d) is our preferred models, shown in Figures 2, 3 and 4 in the main paper. The models show that the location of the low resistivity zones at 20 and 30 km depth are robust, but by reducing the smoothing weights the minimum resistivity reduces from 100 Ω *m* to 10 Ω *m*. Our preferred model is (d) as the overall misfit is significantly better than for all other models.

## Resolution at depth

Data were modelled to a period of 10,000 s, and most of the broadband MT sites had good data to at least 1,000 s. In many crustal settings, such long-period responses would have some sensitivity to the boundary between the lithosphere-asthenosphere boundary. However, in this setting, any potential variations in lithosphere thickness and hence resistivity at depth greater than 100 km are mitigated by much larger variations in resistivity in the top 5 km due to the sediments in the Otway Basin, and the deep ocean to the south, that effectively screens the deeper parts of the Earth.

Supplementary Figure 8 shows the four inversions in Supplementary Figures 6 and 7 for a depth slice at 1 km. The regions in the far southwest is seawater (with a resistivity of 0.3 Ω *m*) and is not shown in the colour scale. More than half the broadband MT sites used in the inversion are on the Otway Basin sediments, that thicken offshore to depths of > 10 km. Thus, most of the bandwidth of MT response is determined by the sediment porosity and seawater. Although sites to the northeast of the array are on more resistive crust (> 1000 Ω *m*), the aperture of the sites (less than 100 by 200 km) yield very little resolution of resistivity variations at depths greater than 100 km. Thus, in Figure 3 of the main paper we do not show resistivity below the LAB as it is un-resolved by the array.

## Additional 2-D modelling

Two-dimensional modelling was undertaken to test whether conductive pathways from the mid-crust to the base of the Otway Basin are a required feature of the data or a model artefact. The data used in the following models are the same as those used in the 3D model presented in the main paper; however, 3D data were first masked in order to avoid 3D effects arising from the use of a 2D inversion algorithm. Dimensionality was determined from skew angle and eccentricity using the freely available MTPy software [38, 39].

Supplementary Figure 9 presents results from 2D inversion along the W70 line (Profile B) of GeoScience Victoria’s 3D Victoria Report 4 [17]. First, a new 2D mesh was constructed with a cell width of 2 km and horizontal padding in both directions of 500 km using a padding factor of 1.5. The mesh has a maximum cell thickness of 400 m down to a depth of 20 km and extends to a maximum depth of 300 km with cell thickness increasing by a factor of 1.2 to 100 km and 1.6 below this point. In total, the mesh for Profile B contains just under 40,000 cells. For Profile C (Supplementary Figure 10), a finer grid was constructed with a cell width of 0.5 km and horizontal padding in both directions of 250 km using a padding factor of 1.5. The mesh has a maximum cell thickness of 250 m down to a depth of 20 km and, from this point, extends down to a maximum depth of 250 km with cell thickness increasing by a factor of 1.2 for a cell count of approximately 64,000 cells.

In both cases, creation of the starting model was the same. First, the new mesh was painted with values interpolated from the 3D model presented in the main paper, after which, all cells above the Proterozoic boundary and below the sedimentary basin were painted to 10,000 Ω m. Two tears were made in the starting model, one along the base of the sedimentary basin and the other along Cambrian/Proterozoic interface. These were incorporated into the model to prevent downward smearing of conductive sediments and upward smoothing of the conductor as a result of regularisation.

Data along each profile were inverted using a period range of 0.001--1000 s and five interpolated points per decade. Inversions were performed using the CGG Geotools™ 2D algorithm and both were stopped after 150 iterations. Profile B achieved a final RMS of 1.305 while Profile C attained an RMS of 0.992.

# References

| [1] | J. Foden, M. A. Elburg, J. Dougherty-Page and A. Burtt, “The timing and duration of the Delamerian Orogeny: correlation with the Ross Orogen and implications for Gondwana assembly, Newer Volcanic Province,” *The Journal of Geology,* vol. 114, p. 189–210, 2006. |
| --- | --- |
| [2] | A. I. S. Kemp, “Petrology of high-Mg, low-Ti igneous rocks of the Glenelg River Complex (SE Australia) and the nature of their interaction with crustal melts,” *Lithos,* vol. 78, p. 119–156, 2004. |
| [3] | P. J. Coney, A. Edwards, R. Hine, F. Morrison and D. Windrim, “The regional tectonics of the Tasman orogenic system, eastern Australia,” *Journal of Structural Geology,* vol. 12, p. 519–543, 1990. |
| [4] | D. A. Foster and D. R. Gray, “Evolution and structure of the Lachlan Fold Belt (Orogen) of Eastern Australia,” *Annual Review of Earth and Planetary Sciences,* vol. 28, p. 47–80, 2000. |
| [5] | R. A. Glen, “The Tasmanides of eastern Australia,” *Geological Society Special Publication,* vol. 246, p. 23–96, 2005. |
| [6] | V. Morand, K. Wohlt, R. Cayley, D. Taylor, A. Kemp, B. Simons and A. Magart, “Glenelg special map area geological report,” 2003. |
| [7] | A. H. M. VandenBerg, “Timing of orogenic events in the Lachlan Orogen,” *Australian Journal of Earth Sciences,* vol. 46, p. 691–701, 1999. |
| [8] | R. A. Cayley, D. H. Taylor, A. H. M. VandenBerg and D. H. Moore, “Proterozoic – Early Palaeozoic rocks and the Tyennan Orogeny in central Victoria: the Selwyn Block and its tectonic implications,” *Australian Journal of Earth Sciences,* vol. 49, p. 225–254, 2002. |
| [9] | R. A. Cayley, R. J. Korsch, D. H. Moore, R. D. Costelloe, A. Nakamura, C. E. Willman, T. J. Rawling, V. J. Morand, P. B. Skladzien and P. J. O'Shea, “Crustal architecture of central Victoria: results from the 2006 deep crustal reflection seismic survey,” *Australian Journal of Earth Sciences,* vol. 58, p. 113–156, 2011. |
| [10] | H. M. V. Stagg, C. D. Cockshell, J. B. Willcox, A. J. Hill, D. V. L. Needham, B. Thomas, G. W. O'Brien and L. P. Hough, Basins of the Great Australian Bight region: geology and petroleum potential, Continental Margins Program ed., vol. A, Australian Government Publishing Service, 1990. |
| [11] | J. B. Willcox and H. M. J. Stagg, “Australia's southern margin: a product of oblique extension,” *Tectonophysics,* vol. 173, p. 269–281, 1990. |
| [12] | D. Perincek and C. D. Cockshell, “The Otway Basin: Early Cretaceous rifting to Neogene inversion,” *The APPEA Journal,* vol. 35, p. 451–466, 1995. |
| [13] | D. S. Edwards, H. I. M. Struckmeyer, M. T. Bradshaw and J. E. Skinner, “Geochemical characteristics of Australia's southern margin petroleum systems,” *The APPEA Journal,* vol. 39, p. 297–321, 1999. |
| [14] | S. Laing, C. N. Dee and P. W. Best, “The Otway Basin,” *The APPEA Journal,* vol. 29, p. 417, 1989. |
| [15] | A. A. Krassay, D. L. Cathro and D. J. Ryan, “A regional tectonostratigraphic framework for the Otway Basin,” 2004. |
| [16] | S. Ryan, L. Knight and G. Parker, “The stratigraphy and structure of the Tyrendarra Embayment, Otway Basin, Victoria, Victorian Initiative for Minerals and Petroleum Report 15, Department of Agriculture,” *Energy and Minerals,* 1995. |
| [17] | P. B. Skladzien, M. A. McLean and T. J. Rawling, *Western Victoria 1:250 000 scale serial sections and accompanying notes. GeoScience Victoria 3D Victoria Report 4. Department of Primary Industries,* 2009. |
| [18] | M. A. McLean, G. A. Pears, M. Boyd and R. A. Cayley, *Quantitative interpretation of basement structure using 3D inversion modelling of airborne gravity data. VGP Technical Report 63. Geological Survey of Victoria, Department of Jobs, Precincts and Regions, Melbourne, Victoria.,* 2021. |
| [19] | M. Mclean, P. Skladzien, B. Williams and T. Rawling, *Glenelg/Grampians-Stavely Zone 1:250 000 scale 3D geological fault model metadata notes. GeoScience Victoria 3D Victoria Report 6. Department of Primary Industries,* 2010. |
| [20] | S. J. Holt, S. P. Holford and J. Foden, “New insights into the magmatic plumbing system of the South Australian Quaternary Basalt province from 3D seismic and geochemical data,” *Australian Journal of Earth Sciences,* vol. 60, p. 797–817, 2013. |
| [21] | A. R. Chivas, I. Barnes, W. C. Evans, J. E. Lupton and J. O. Stone, “Liquid carbon dioxide of magmatic origin and its role in volcanic eruptions,” *Nature,* vol. 326, p. 587–589, 1987. |
| [22] | M. W. Caffee, G. B. Hudson, C. Velsko, E. C. Alexander Jr, G. R. Huss and A. R. Chivas, “Non-atmospheric noble gases from CO2 well gases,” in *19ᵗʰ Lunar and Planetary Science Conference*, 1988. |
| [23] | W. F. Giggenbach, Y. Sano and H. U. Schmincke, “CO2-rich gases from Lakes Nyos and Monoun, Cameroon; Laacher See, Germany; Dieng, Indonesia, and Mt. Gambier, Australia—variations on a common theme,” *Journal of Volcanology and Geothermal Research,* vol. 45, p. 311–323, 1991. |
| [24] | K. Chatfield, “The relationship between volcanics, associated intrusives and carbon dioxide within the Otway Basin, South Australia,” 1992. |
| [25] | P. W. J. Glover, “Graphite and electrical conductivity in the lower continental crust: A review,” *Physics and Chemistry of the Earth,* vol. 21, p. 279–287, 1996. |
| [26] | L. Dai, H. Li, H. Hu, S. Shan, J. Jiang and K. Hui, “The effect of chemical composition and oxygen fugacity on the electrical conductivity of dry and hydrous garnet at high temperatures and pressures,” *Contributions to Mineralogy and Petrology,* vol. 163, p. 689–700, September 2012. |
| [27] | D. Wang, Y. Guo, Y. Yu and S.-i. Karato, “Electrical conductivity of amphibole-bearing rocks: influence of dehydration,” *Contributions to Mineralogy and Petrology,* vol. 164, p. 17–25, 2012. |
| [28] | R. R. Hillis, S. A. Monte, C. P. Tan and D. R. Willoughby, “The contemporary stress field of the Otway Basin, South Australia: implications for hydrocarbon exploration and production,” *APEA Journal,* vol. 35, 1995. |
| [29] | M. Sandiford, “Geomorphic constraints on the Late Neogene tectonics of the Otway Range, Victoria,” *Australian Journal of Earth Sciences,* vol. 50, p. 69–80, February 2003. |
| [30] | M. Sandiford, M. Wallace and D. Coblentz, “Origin of the in situ stress field in south-eastern Australia,” *Basin Research,* vol. 16, p. 325–338, September 2004. |
| [31] | S. J. Orfanidis, Introduction to signal processing, Prentice-Hall, Inc., 1995. |
| [32] | T. G. Caldwell, H. M. Bibby and C. Brown, “The magnetotelluric phase tensor,” *Geophysical Journal International,* vol. 158, p. 457–469, 2004. |
| [33] | A. Kirkby, G. Heinson, S. Holford and S. Thiel, “Mapping fractures using 1D anisotropic modelling of magnetotelluric data: a case study from the Otway Basin, Victoria, Australia,” *Geophysical Journal International,* vol. 201, p. 1961–1976, 4 2015. |
| [34] | W. D. Parkinson, “The influence of continents and oceans on geomagnetic variations,” *Geophysical Journal International,* vol. 6, p. 441–449, 1962. |
| [35] | G. Heinson, J. Duan, A. Kirkby, K. Robertson, S. Thiel, S. Aivazpourporgou and W. Soyer, “Lower crustal resistivity signature of an orogenic gold system,” *Nature Geoscience,* 2021. |
| [36] | G. D. Egbert and A. Kelbert, “Computational recipes for electromagnetic inverse problems,” *Geophysical Journal International,* vol. 189, p. 251–267, 1 2012. |
| [37] | K. Robertson, S. Thiel and N. Meqbel, “Quality over quantity: on workflow and model space exploration of 3D inversion of MT data,” *Earth, Planets and Space,* vol. 72, p. 1–22, 2020. |
| [38] | L. Krieger and J. R. Peacock, “MTpy: A Python toolbox for magnetotellurics,” *Computers & Geosciences,* vol. 72, p. 167–175, November 2014. |
| [39] | A. Kirkby, F. Zhang, J. Peacock, R. Hassan and J. Duan, “The MTPy software package for magnetotelluric data analysis and visualisation,” *Journal of Open Source Software,* vol. 4, p. 1358, May 2019. |
| [40] | M. Hall and J. Keetley, “Otway Basin: stratigraphic and tectonic framework. GeoScience Victoria 3D Victoria Report 2,” 2009. |
| [41] | R. J. Willink and R. Lovibond, “Technology, teamwork, respect and persistence: ingredients of successful exploration in the onshore otway basin,” *The APPEA Journal,* vol. 41, p. 53, 2001. |
| [42] | C. Lesti, G. Giordano, F. Salvini and R. Cas, “Volcano tectonic setting of the intraplate, Pliocene-Holocene, Newer Volcanic Province southeast Australia: role of crustal fracture zones,” *Journal of Geophysical Research,* vol. 113, July 2008. |
| [43] | S. Aivazpourporgou, S. Thiel, P. C. Hayman, L. N. Moresi and G. Heinson, “Decompression melting driving intraplate volcanism in Australia: Evidence from magnetotelluric sounding,” *Geophysical Research Letters,* vol. 42, p. 346–354, 2015. |


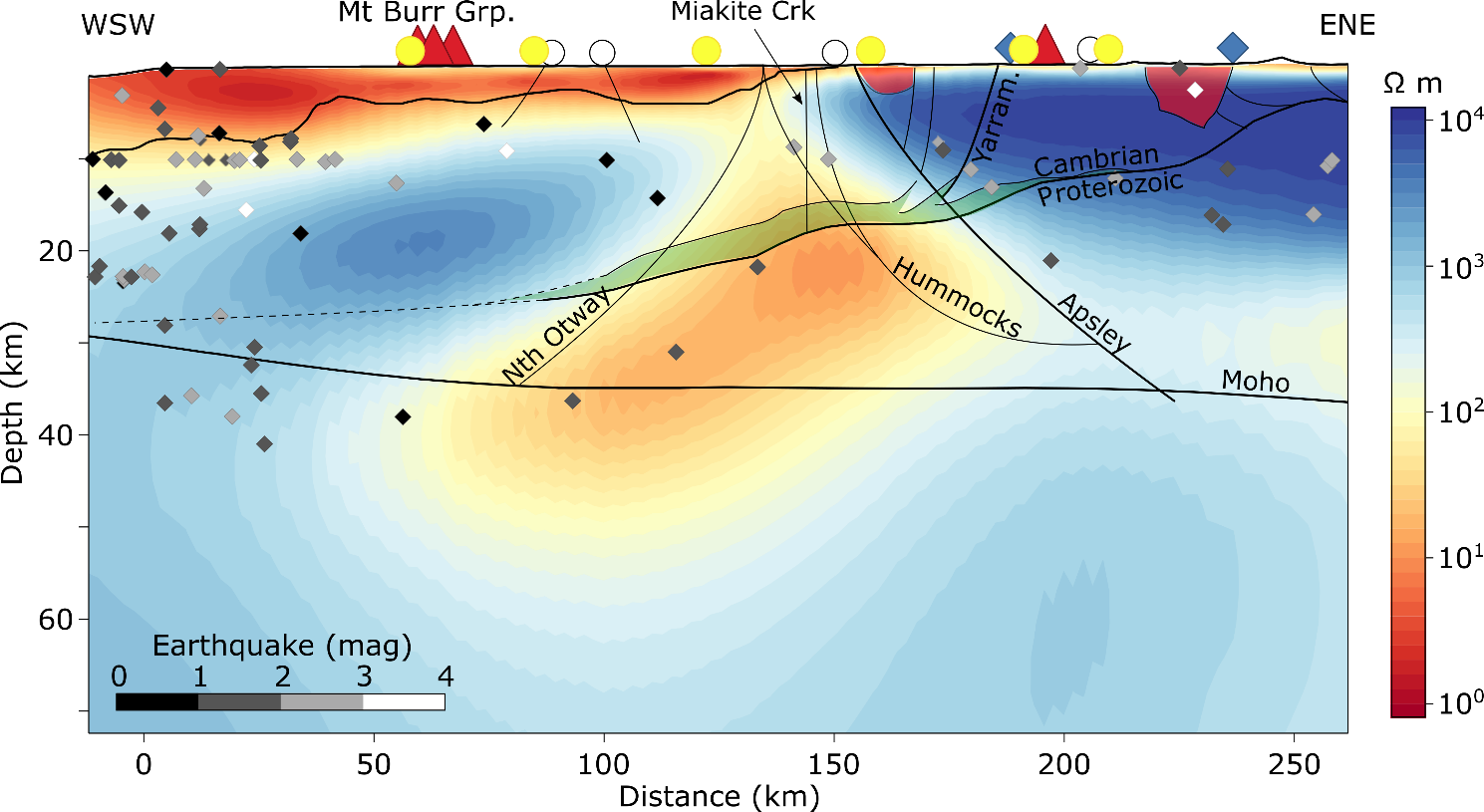


Supplementary Figure 1 - Profile B: Resistivity cross-section (2x vertical exaggeration) along the W70 line from GeoScience Victoria’s 3D Victoria Report 4 [17]. The green layer is an oceanic crust/mantle layer inferred by the authors of 3D Victoria Report 4 and known granitic plutons are in red. Simplified structural representation includes the well-established Miakite Creek, Apsley, Hummocks and Yarramyljup fault systems and is based on previous seismic and gravitational modelling within the region [17, 8, 40]. The location of Profile B can be seen in Supplementary Figure 5. Yellow circles: broadband MT sites as part of this paper, white circles: previously collected broadband MT, blue diamonds: AusLAMP and other long-period MT.


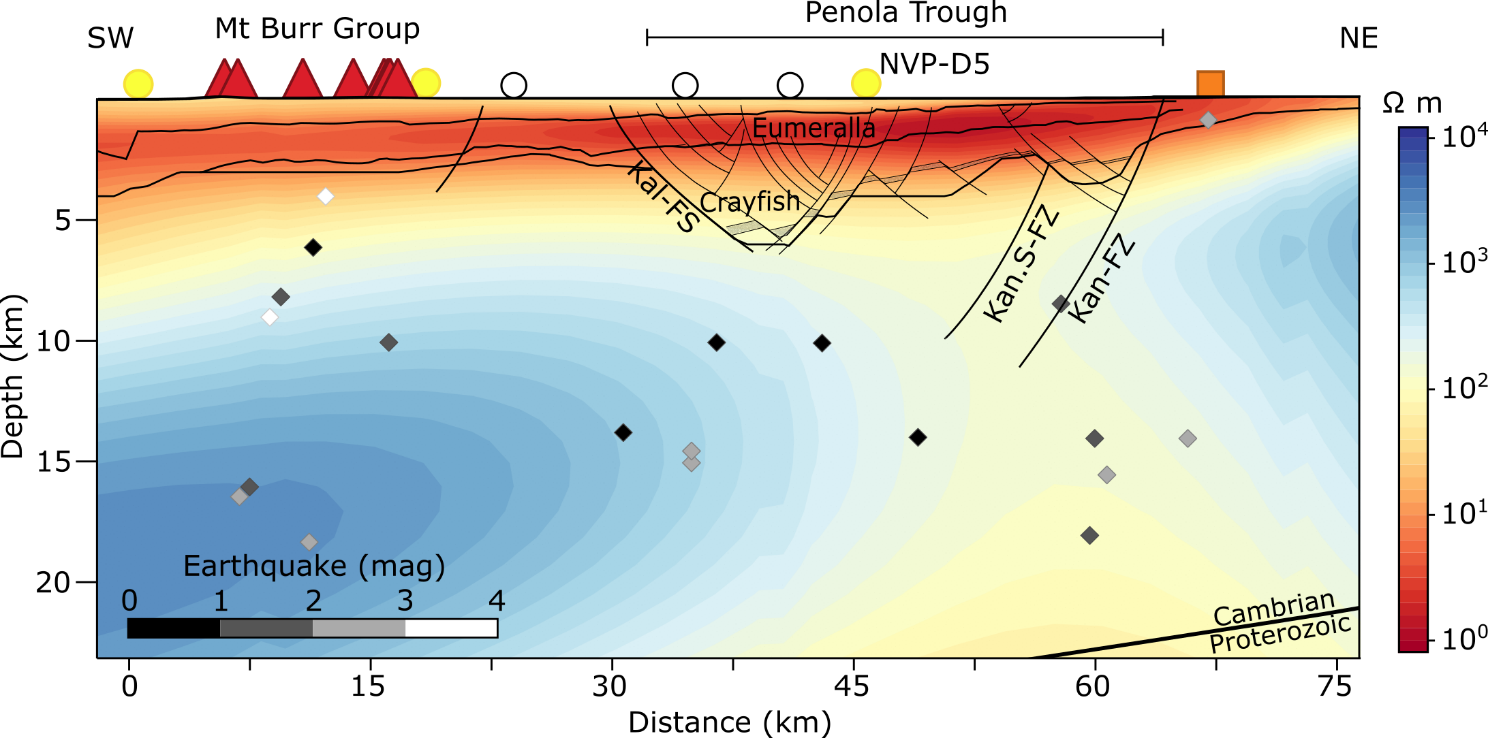


Supplementary Figure 2 - Profile C*:* Resistivity section across the Penola Trough showing the conductive pathway through the upper crust to the base of the Otway Basin. The Penola trough is bounded in the SW by the Kalangadoo Fault System and in the NE by the Kanawinka South and Kanawinka Fault System. Volcanoes of the Mt Burr Group are displayed as red triangles. Structural interpretation based on previous research incorporating results from seismic, magnetics and gravity modelling [41, 17, 9]. The location of Profile B can be seen in Supplementary Figure 5 Yellow circles: broadband MT sites as part of this paper, white circles: previously collected broadband MT, orange squares: GDS sites.


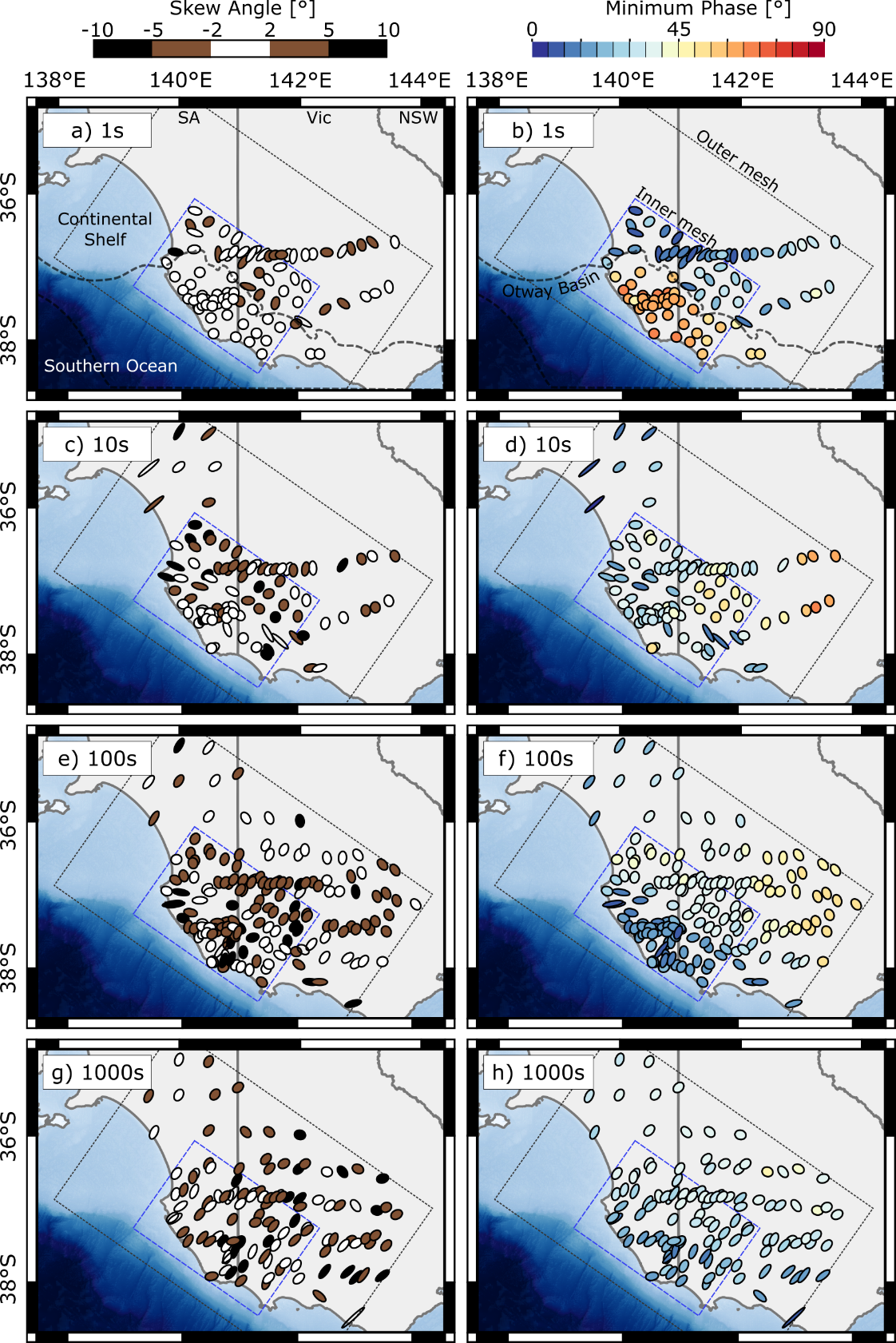


Supplementary Figure 3 - Skew angle (left) and phase minima (right) at various periods for modelled MT sites across the Mt Gambier subprovince. Some data were rejected from modelling due to poor quality and appear as gaps in these images. In most cases, missing data was accounted for by incorporating nearby sites from previous surveys. The small dashed rectangle is equivalent to the inner starting mesh of the 3D model and the dark dotted line represents the outer mesh. All newly collected broadband MT sites are located within the inner mesh.


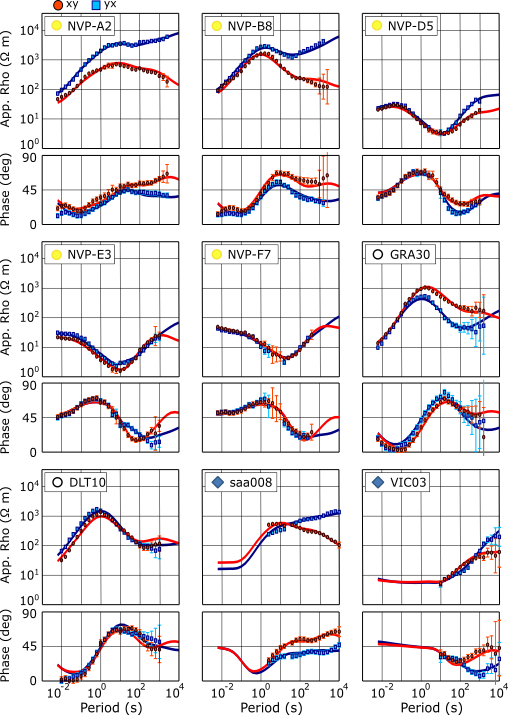


Supplementary Figure 4 - Select MT soundings for key sites across across the survey area. Solid lines represent the modelled fit to the data. Locations for each plot can be seen in Supplementary Figure 5


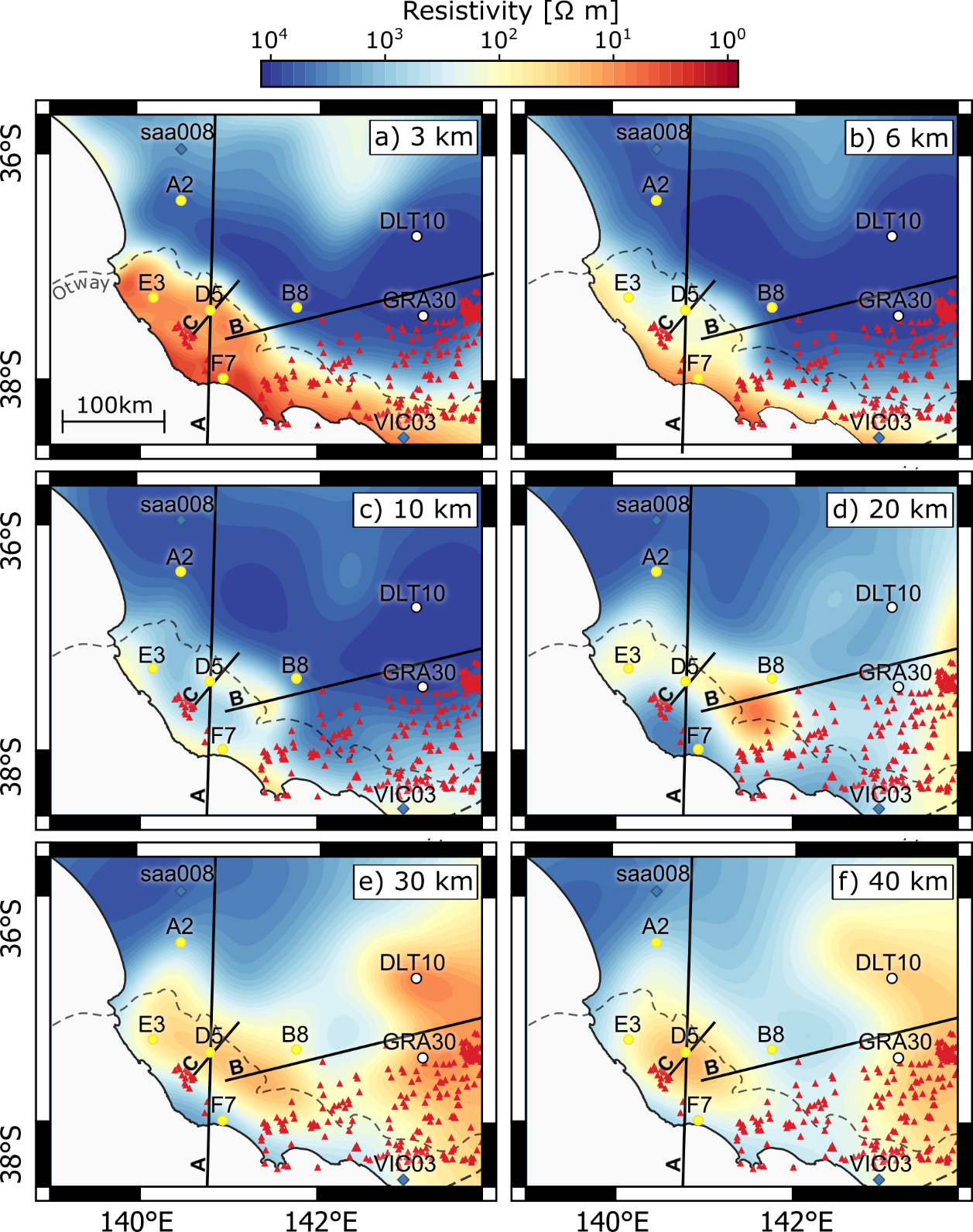


Supplementary Figure 5 - Additional resistivity depth slices across the Mt Gambier region. Profiles A, B and C are illustrated by solid black lines. The labelled markers represent a select number of sites chosen to illustrate data quality and model fits and various points in the model. The grey area to the west represents ocean and is masked from the figure as there are no data to constrain the model beyond the coastline. At 3km depth, the conductive feature is representative of conductive sediments of the Otway Basin (see Supplementary Figure 2 for a profile view of this feature). The large conductor visible to the east at depths below ~30km is a separate feature and has been discussed previously [35, 43].


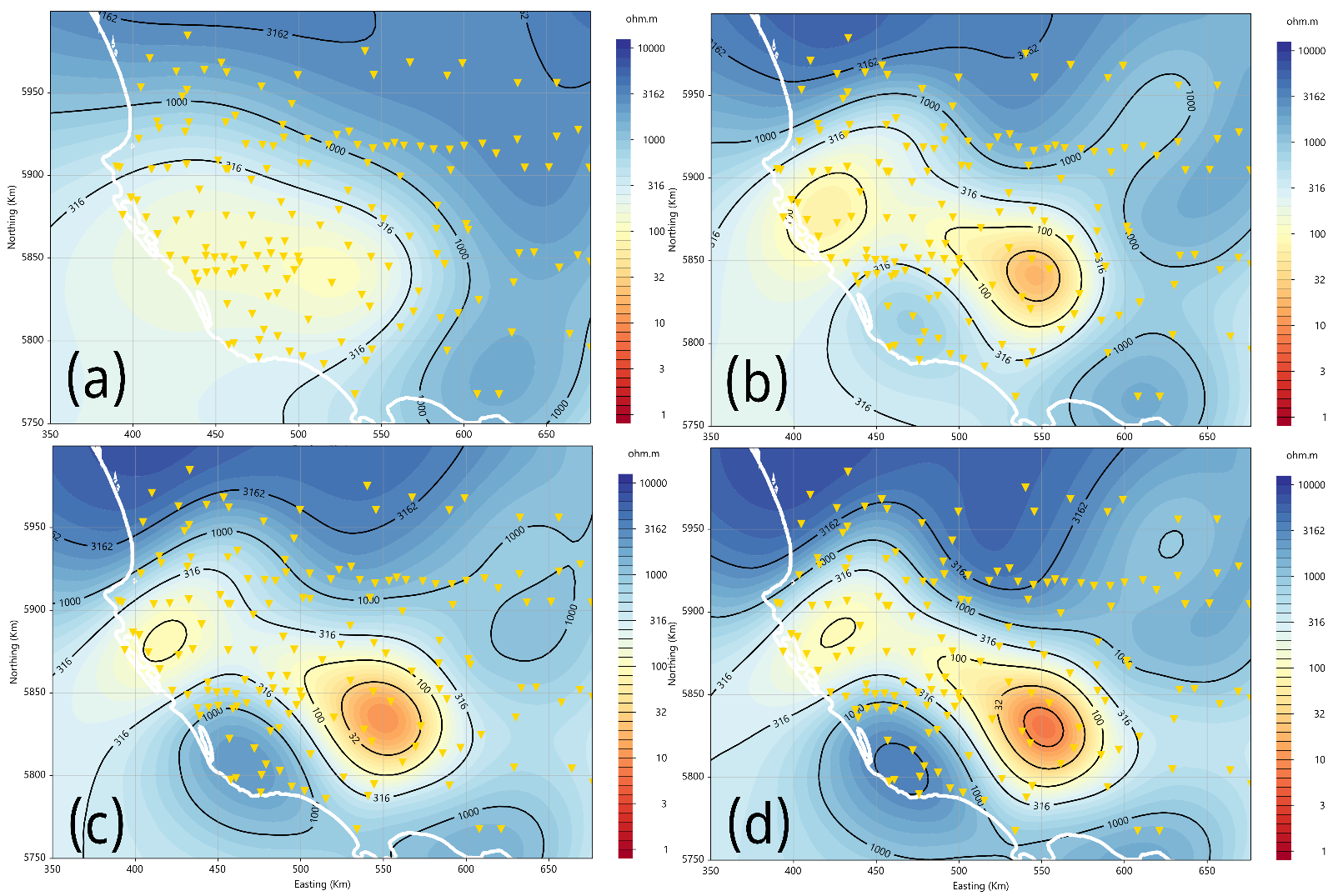


Supplementary Figure 6 - Plan views of the 3D resistivity models at a depth of 20 km in grid projection UTM Zone 54S. The white line shows the coastline, and the yellow triangles are the MT sites used in the inversion. Models (a) to (d) have progressive reduction in smoothing weights


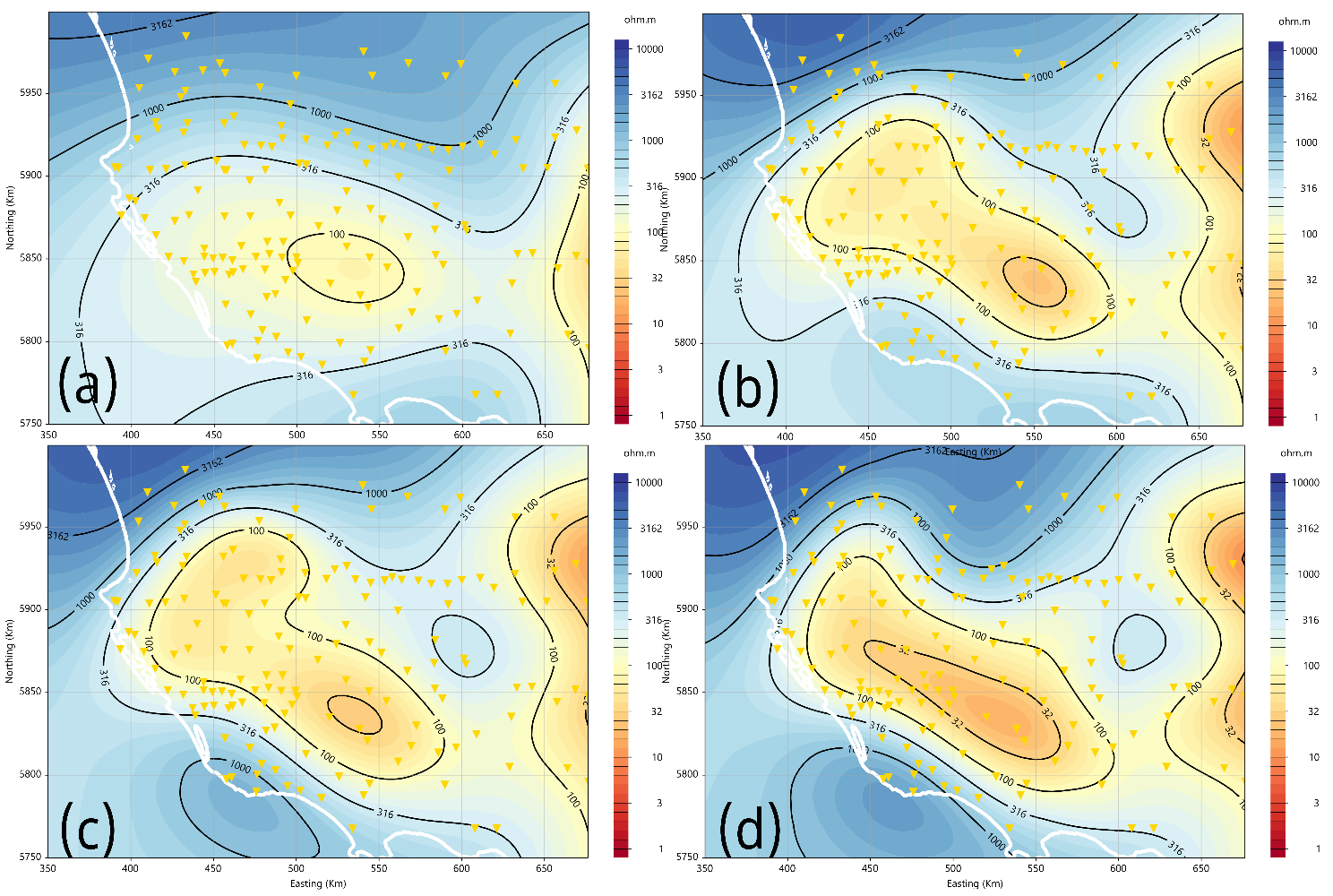


Supplementary Figure 7 - Plan views of the 3D resistivity models at a depth of 30 km in grid projection UTM Zone 54S. The white line shows the coastline, and the yellow triangles are the MT sites used in the inversion. Models (a) to (d) have progressive reduction in smoothing weights


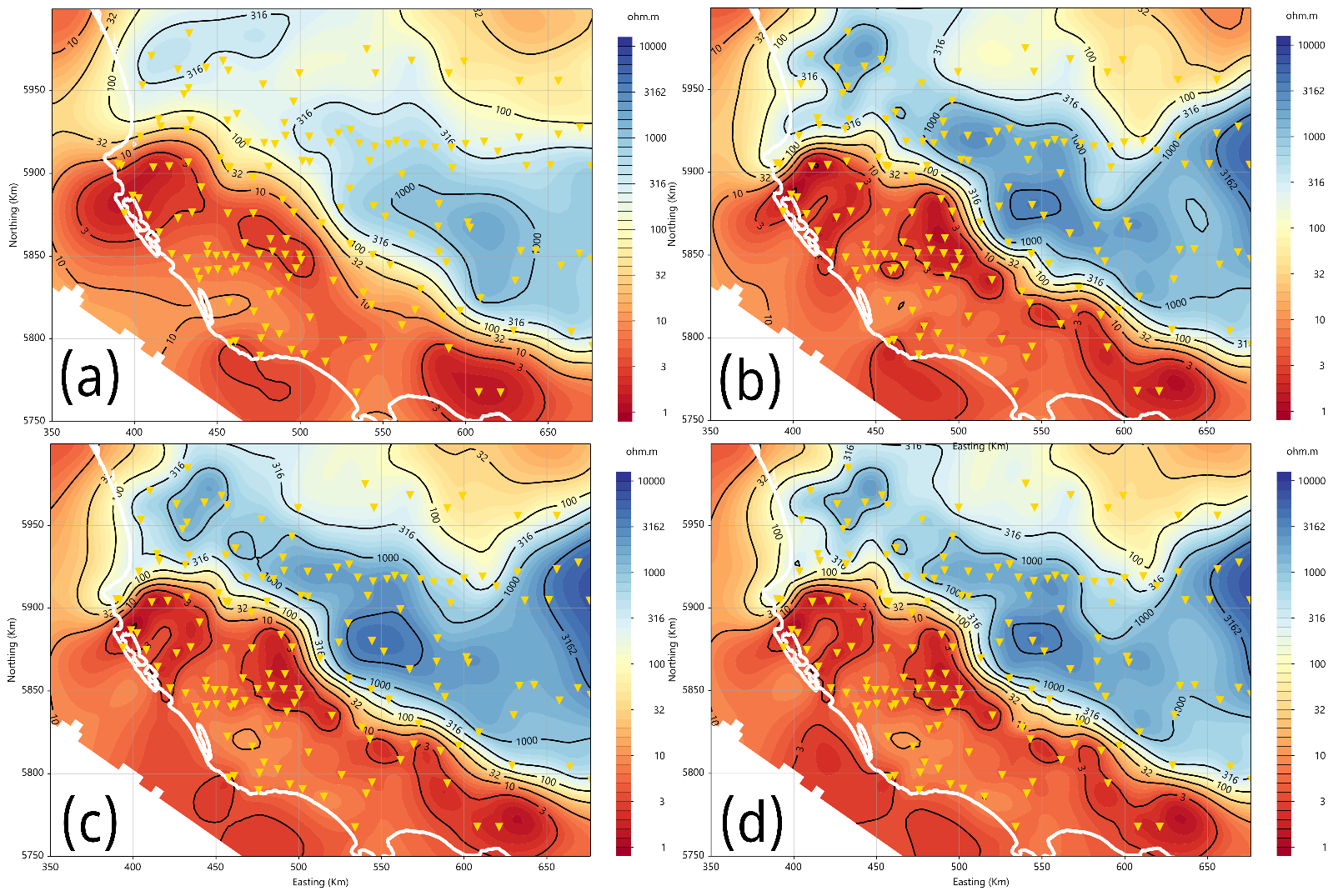
Supplementary Figure 8 - Plan views of the 3D resistivity models at a depth of 1 km in grid projection UTM Zone 54S. The white line shows the coastline, and the yellow triangles are the MT sites used in the inversion. Models (a) to (d) have progressive reduction in smoothing weights. All models show the significant variation in resistivity from the Otway Basin (< 3 Ω m) and the basement rocks to the northeast (>1000 Ω m). In the most northeast corner, the low resistivity is due to sediments of the Murray Basin.


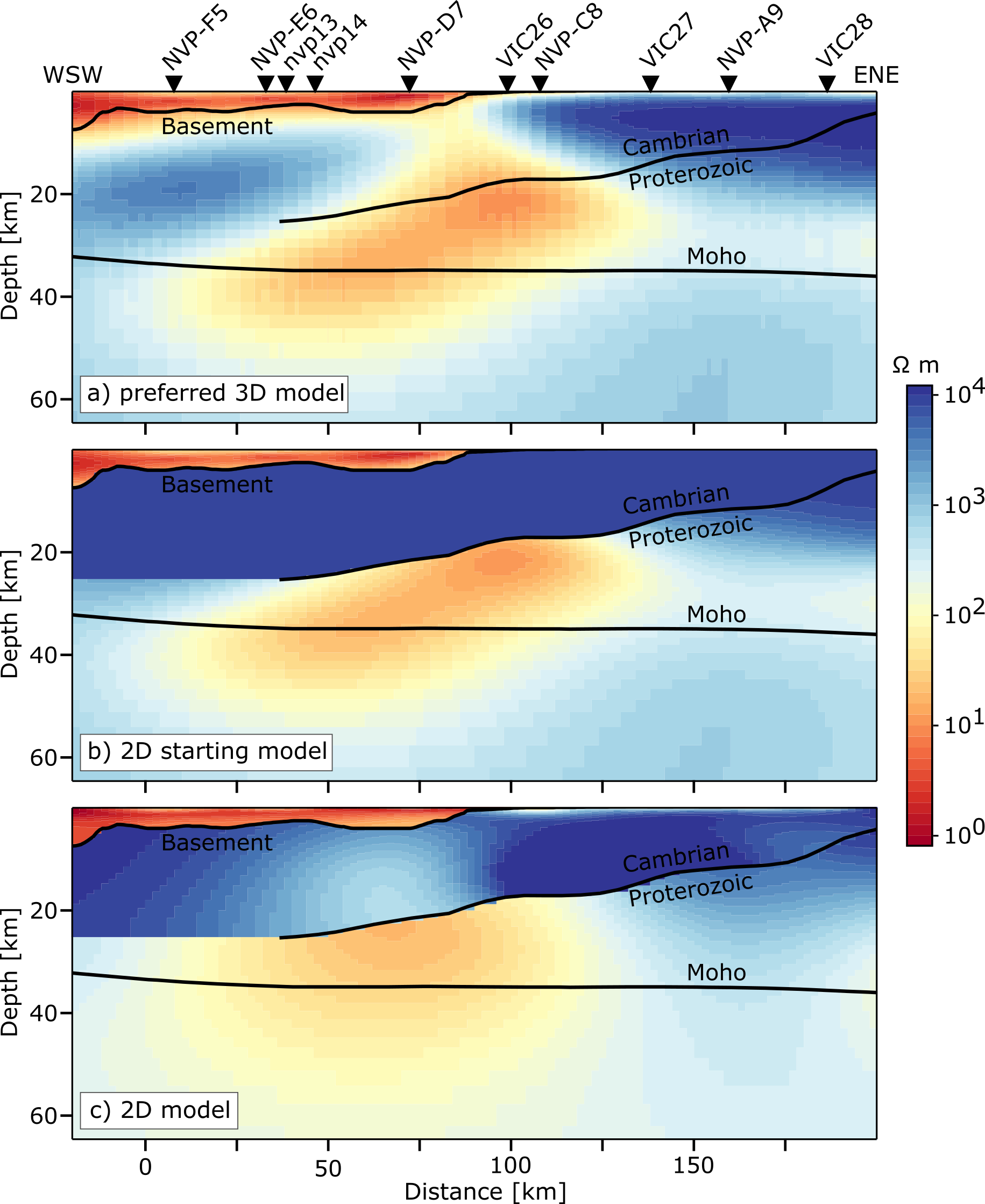


Supplementary Figure 9 - Results from 2D inversion along Profile B (see Figure 2). a) the preferred 3D model from the main paper, b) the starting model used for the 2D inversion. All cells between the Cambrian/Proterozoic interface and the bottom of the sedimentary basin were assigned a value of 10,000 Ω m and a tear was made along both surfaces to prevent smearing. c) Output from the 2D inversion showing a preference for a conductive pathway below site NVP-D7.


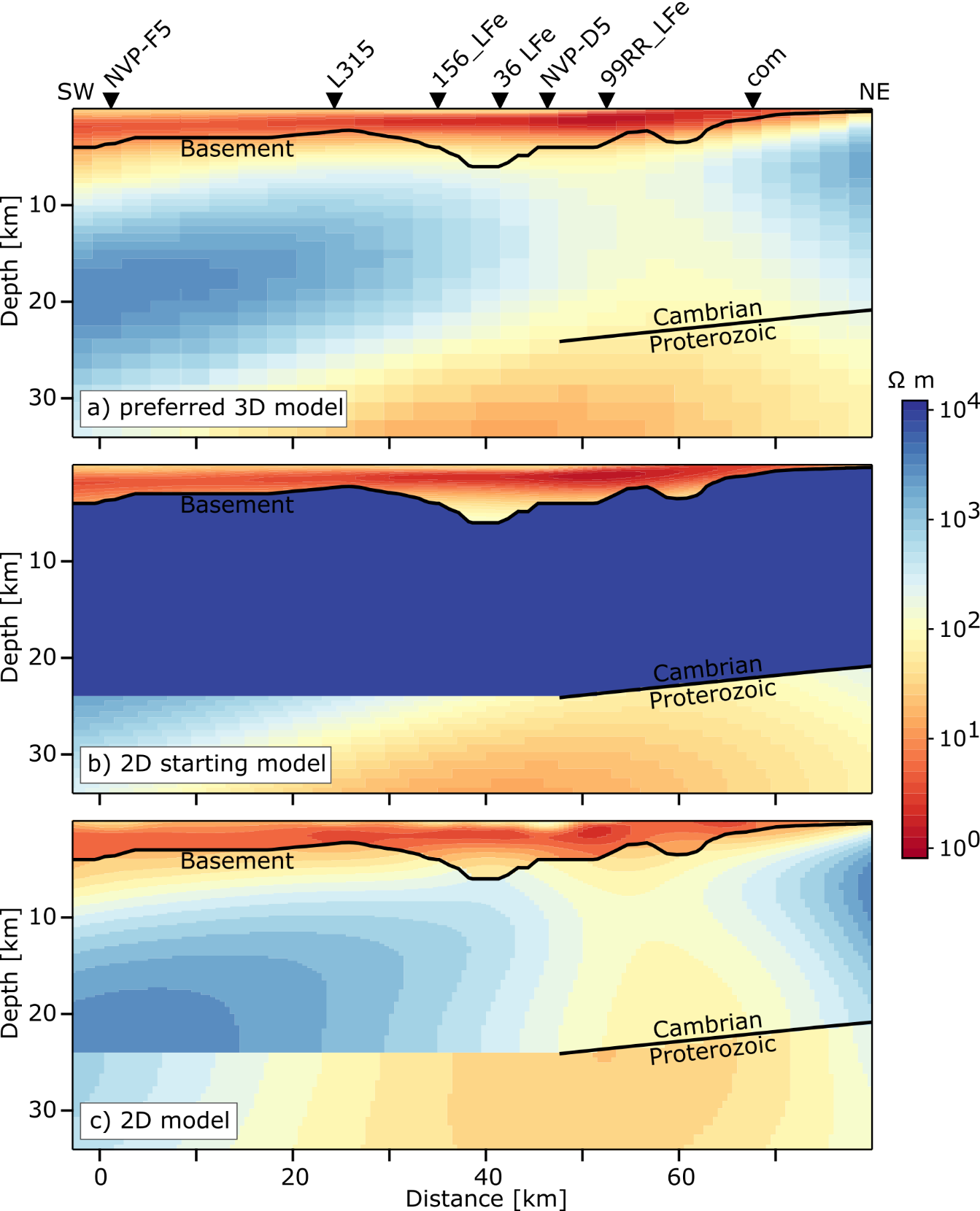


Supplementary Figure 10 - Results from 2D inversion along Profile C (see Figure 2). a) the preferred 3D model from the main paper, b) the starting model used for the 2D inversion. All cells between the Cambrian/Proterozoic interface and the bottom of the sedimentary basin were assigned a value of 10,000 Ω m and a tear was made along both surfaces to prevent smearing. c) Output from the 2D inversion showing a preference for a conductive pathway slightly northeast of site NVP-D5.
